# The surgical outcomes of trans-scaphoid perilunate fracture-dislocations

**DOI:** 10.3906/sag-1710-163

**Published:** 2020-02-13

**Authors:** Cemal KURAL, Bülent TANRIVERDİ, Ersin ERÇİN, Emre BACA, Murat YILMAZ

**Affiliations:** 1 Clinic of Orthopaedics and Traumatology, Bakırköy Dr. Sadi Konuk Research and Training Hospital, İstanbul Turkey2; 2 Clinic of Orthopaedics and Traumatology, Haseki Research and Training Hospital, İstanbul Turkey

**Keywords:** Dislocation, open reduction, perilunate, scaphoid

## Abstract

**Background/aim:**

Trans-scaphoid perilunate fracture-dislocation (TSPFD) is a rare injury. TSPFD is a fracture-dislocation that severely disrupts the anatomical structure of the carpal bones and may occur as a result of a high energy trauma of the wrist or a fall on an open hand. In this study, the aim is to provide midterm clinical and radiological evaluations of cases diagnosed and treated as TSPFD.

**Materials and methods:**

Eleven patients diagnosed with TSPFD as a result of wrist trauma were treated surgically and were analysed retrospectively. Clinical and radiological follow-up of the cases was evaluated. The mean age of the patients was 34 years. All patients were males with a dorsal dislocation according to Herzberg’s perilunate fracture-dislocation classification. The mean follow-up time was 33 months. All of the cases were evaluated with preoperative and postoperative standard wrist anteroposterior and lateral radiographs. A dorsal approach was used in all cases. However, in 1 case a volar approach was also required. The Green and O’Brien evaluation scale modified by Cooney was used for the clinical assessment of pain, wrist range of motion, grip strength, and functional status as excellent, good, moderate, or poor. The wrist range of motion was evaluated goniometrically at the final check-up, and a mid-grade disability was observed compared with the uninjured side. A visual analogue scale was used to evaluate the pain.

**Results:**

Sufficient union was obtained in all cases with open reduction and internal fixation of the fractures. Grip strength was up to 77.5% of the other side. According to the modified Green and O’Brien clinical evaluation scale, 6 cases were evaluated as good, 3 cases were fair, and 2 cases were poor. No median nerve damage was determined preoperatively or postoperatively and there was no postoperative pin tract infection in any of the patients.

**Conclusion:**

This kind of injury represents complex biomechanical damage of the wrist anatomy. If it is diagnosed early and treated with open reduction and stable fixation, a functionally adequate and anatomically integrated wrist can be achieved.

## 1. Introduction

Trans-scaphoid perilunate fracture-dislocation (TSPFD) is a rare injury. TSPFD is a fracture-dislocation that severely disrupts the anatomical structure of the carpal bones. Perilunate dislocations represent 7% of carpal area injuries [1]. Of acute perilunate dislocations, 65% are dorsal TSPFDs [2]. This injury is characterized by the dorsal dislocation of the capitate head from the distal lunate joint [3]. However, palmar dislocations are rarely encountered.

This kind of injury typically occurs as a result of a high energy trauma of the wrist or a fall on an open hand [4]. Due to a missed or incorrect diagnosis, or ignorance of these severe wrist injuries, there is a delay in treatment in 1/4 of the cases [5]. This injury is divided into 3 groups according to the length of time between the trauma and the treatment. The first week after the injury is the acute phase, 7 to 45 days after the injury is referred to as the neglected phase, and >45 days is the chronic phase [2]. Treatment may consist of closed reduction and cast immobilization, open reduction and internal fixation, ligament repair, limited wrist arthrodesis, or, as a final salvage, a proximal row carpectomy, depending on the time of diagnosis and any additional pathological findings [6].

The objective of this study is to provide midterm clinical and radiological evaluations of cases diagnosed and treated as TSPFD.

## 2. Materials and methods

There were 11 cases diagnosed as TSPFD as a result of wrist trauma and treated surgically at 2 training and research hospitals that were analysed retrospectively. The clinical and radiological follow-up of the cases was evaluated. All patients were males with a dorsal dislocation. While all of the cases were the result of falling in terms of trauma aetiology, a detailed analysis indicated that 2 cases were occupational accidents, 2 cases occurred during participation in sports activities, 1 case was a suicide attempt by a patient diagnosed with schizophrenia, and 6 cases were simple falls (Table 1).

**Table 1:** Trauma aetiology, accompanying injuries, and postoperative evaluation results.

Patient	Accompanying injuries	Aetiology	Ext./flex.(degree)	Normal sideext./flex.(degree)	Gripstrength	Wrist degeneration (radiological evaluation)	Clinical assessment(modified Greenand O’Brien)
1	-Acetabulum-Prox. Femur	Work accident	30/35	80/85	70	Stage 4	Good
2	-Pubis	Fall	50/30	80/70	70	Stage 2	Fair
3	-Olecranon-Radial head	Fall during sports	20/50	80/80	80	Stage 2	Poor
4	-Calcaneus	Suicide attempt	40/50	80/90	80	Stage 1	Good
5	-Distal radius	Fall	50/30	90/90	70	Stage 1	Fair
6	-Trochanter major	Fall during sports	40/30	80/85	75	Stage 2	Good
7	-	Fall	25/30	85/85	70	Stage 2	Fair
8	-Pilon-Calcaneus-Vertebra	Work accident	45/45	80/80	90	Stage 4	Poor
9	-Mandibula-Maxilla	Fall	40/70	80/80	90	Stage 3	Good
10	-	Fall	40/55	80/80	80	Stage 1	Good
11	-	Fall	40/40	80/80	80	Stage 2	Good

Ext: Extension, Flex: Flexion, Prox: Proximal.

The mean patient age was 34 years (range: 23–46 years). Three of the 11 patients had a delayed diagnosis: 2 cases were first diagnosed as elbow injuries from a simple fall and treated with cast, then later assessed as TSPFD at the first control visit. One patient had multiple trauma; his femur and acetabulum fractures were treated initially, but he subsequently complained about wrist pain and was diagnosed with TSPFD. These delayed phase patients were respectively treated surgically on the 10th, 11th, and 27th day after the injury. The remaining 8 cases were diagnosed correctly at the first assessment and treated surgically within 1 to 5 days as acute cases (Table 2).

**Table 2:** The preoperative and postoperative follow-up details.

Patients	Age(years)	Type of perilunate dislocation	Time from trauma to surgery (days)	Surgical approach	Postoperative cast duration (weeks)	Follow-up duration(months)
1	46	T-S	10	Dorsal+volar	12	15
2	29	T-S	27	Dorsal	6	13
3	42	T-S	3	Dorsal	6	30
4	32	T-S	5	Dorsal	6	26
5	29	T-S	3	Dorsal	8	25
6	28	T-RS-S	2	Dorsal	8	18
7	41	T-S	3	Dorsal	6	32
8	23	T-S-C	11	Dorsal	6	56
9	38	T-RS-S	1	Dorsal	6	14
10	42	T-S	4	Dorsal	6	62
11	29	T-S	2	Dorsal	8	13

T-RS-S: Trans-radialstyloid, trans-scaphoid; T-S: Trans-scaphoid; T-S-C:Trans-scaphoid, trans-capitate.

Only 3 of the cases did not have an additional injury; the other 8 cases had various accompanying extremity injuries (Table 1). Eight cases were trans-scaphoid, 2 cases were trans-radial styloid and trans-scaphoid, and 1 case was trans-scaphoid and trans-capitate. 

All of the cases were evaluated with preoperative and postoperative standard wrist anteroposterior (AP) and lateral (LAT) radiographs. According to Herzberg’s perilunate fracture-dislocation classification [7], all of the cases were of the dorsal type. Four cases were determined to be stage 1 and 7 cases were stage 2. All of the cases were scaphoid body fractures, and in addition, 2 had triquetrum fractures, 2 had distal ulna styloid fractures, 2 cases had distal radius styloid fractures, and 1 had a capitatum fracture.

**Figure 1:**
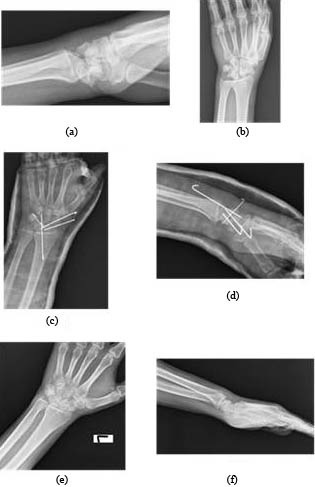
The radiographs of patient 4, a 32-year-old man who is schizophrenic, injured in a fall from a height in a suicide attempt: a) The preoperative trans-scaphoid perilunate fracturedislocation lateral X-ray, b) Anteroposterior (AP) X-ray, c) The postoperative AP view after open reduction and internal fixation with K wires, d)The postoperative lateral view, e) AP X-ray at 26-month control, f) The lateral view at 26-month control.

**Figure 2:**
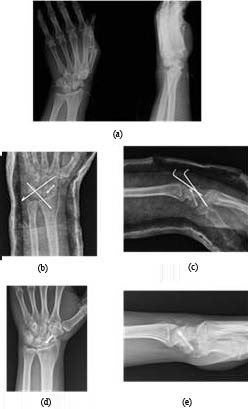
The radiographs of patient 3, a 42-year-old man, injured in a fall: a) The preoperative trans-scaphoid perilunate fracture-dislocation anteroposterior (AP) and lateral X-rays, b) The postoperative AP view after open reduction internal fixation with Herbert screws and K wires, c) The postoperative lateral view, d) AP X-ray at 30-month control, e) The lateral view at 30-month control.

A dorsal approach was used in all cases. However, in 1 case a volar approach was also required. In that case, the palmar ligament was repaired as well. For internal fixation of the scaphoid fractures, Kirschner (K) wires were used in 5 cases (Figure 1), headless screws were used in 4 cases, and Herbert screws were used in 2 cases (Figure 2). C-arm fluoroscopy was used to examine the instability, and the capitolunate and lunotriquetral joints were stabilized with K wires. The other fractures were stabilized with 1.8 mm K wires. A graft was not required in any of the cases. The K wires used for scaphoid fractures were removed in 8 to 10 weeks, and the K wires used for the other fractures were removed in 5 to 6 weeks. In all cases, a cast with a thumb spica was applied postoperatively. Additional AP, LAT, and oblique radiographs were used to evaluate the fracture union. Cross trabeculation was assessed [8]. A goniometer was used to evaluate the wrist range of motion, and a dynamometer was used to evaluate the grip strength. 

The wrist degeneration was evaluated by X-ray and mild changes in the radial styloid were evaluated as stage 1; degeneration in the radial and scaphoid joint surfaces was evaluated as stage 2; degeneration in radioscaphoid and capitolunate was evaluated as stage 3; and degeneration in the radiocarpal and intercarpal joints was evaluated as stage 4 [9]. The Green and O’Brien evaluation scale modified by Cooney was used for the clinical assessment of pain, wrist range of motion, grip strength, and functional status as excellent, good, moderate, or poor [10]. A visual analogue scale (VAS) was used to evaluate the pain.

After removal of the fixation materials and cast, patients began a physical rehabilitation program of muscle strengthening and range of motion for the hand and the wrist. Participation in sports and other activities was allowed after 5 to 6 months.

As a statistical method, mean, minimum, and maximum values ​​were given for the continuous variables. Spearman correlation analysis was used to determine the correlational relationships between the variables. P < 0.05 was considered statistically significant. Analyses were performed with NCSS 11 (Number Cruncher Statistical System, 2017 Statistical Software, Utah, USA).****

## 3. Results

Sufficient union was obtained in all of these cases treated with open reduction and internal fixation of the fractures. No reduction loss, pseudarthrosis, or avascular necrosis was observed.

The mean follow-up time was 33 months (range: 19–68 months). In terms of wrist range of motion, a mid-grade disability (average: 38°) was observed compared with the uninjured side. The grip strength was up to 77.5% of the other side (Table 3). The radiological assessment of the wrist degeneration indicated that 2 cases were stage 4, 1 was stage 3, 5 cases were stage 2, and 3 cases were stage 1 (Table 1).

According to the modified Green and O’Brien clinical evaluation scale, 6 cases were evaluated as good, 3 cases were fair, and 2 cases were poor. One of the 2 poor cases was a delayed case, while the other was an acute case.

Nine of our patients returned to their former jobs. One patient who had olecranon and radial head fractures and 1 patient who had pilon-calcaneus and vertebral fractures had to change their jobs due to the wrist and ankle pain. The mean VAS score was 2.9 (range: 2–4) in pain evaluation.

Median nerve damage was not determined in the preoperative or postoperative period in any of the patients. There was no postoperative pin tract infection in any of the patients.

Statistically, there was no correlation between the wrist degeneration stage and the range of motion, the VAS score, and the grip strength. There was a negative correlation between the VAS score and the wrist degeneration level (P = 0.049, rho = –0.603).

## 4. Discussion

In this study, the clinical and radiological follow-up results of 11 patients with TSPFD, which is a rare injury, are presented. A perilunate injury is rare among the wrist traumas [2]. In perilunate dislocations resulting from severe isolated ligament injuries, the capitate and remainder of the carpus dislocate around the lunate in a dorsal or palmar direction. A dorsal perilunate dislocation is more common (90%). Rarely, the connection between the lunate and the radius is ruptured and the lunate bone is displaced dorsally or palmarly while the radial connection to the other carpal bones is protected. This is a lunate dislocation. Scaphoid fractures are associated with 60% of perilunate or lunate dislocations; this kind of injury is called a perilunate fracture-dislocation. TSPFD are often accompanied by radial styloid, capitatum, or triquetrum fractures [7,10].

The diagnosis of carpal fracture-dislocations is often missed initially [7]. Given that the patient often has other injuries that demand the physician’s immediate attention and if the radiological images are not appropriate, the diagnosis may be delayed. Eight of our patients had injuries to other parts of the body. In 2 cases the TSPFD was not diagnosed by X-ray until 7 to 10 days after the injury. Another patient who was also treated for other injuries was diagnosed by X-ray 3 weeks after the injury and was sent to our hospital as a delayed case.

As cited by Gellman et al., in 1959 Wagner proposed arthrodesis to treat a TSPFD, if closed reduction was not possible; in 1965 Campbell recommended proximal row carpectomy; and in 1944 MacAusland proposed open reduction if diagnosed within 6 weeks and lunate excision for the cases diagnosed later [11]. Some previous studies proposed an open reduction and internal fixation even in delayed cases [4,11]. Reduction loss and instability often occur in cases diagnosed as acute TSPFD due to scaphoid fracture even when closed reduction is achieved [12,13]. 

Adkison and Chapman identified a 59% reduction loss in 6 weeks in 55 cases with TSPFD treated with closed reduction and cast immobilization [14]. For pain relief and reduction, traction and reduction manoeuvres are performed in the emergency room in this kind of case. However, this can be very difficult, especially when the lunate is rotated and dislocated palmarly. Such manoeuvres may even exacerbate palmar radiolunate ligament injuries [14]. 

Open reduction allows for a better evaluation of the injury, as well as reduction and repair [6]. In terms of the surgical approach, a volar, dorsal, or combined approach may be used. The decision must be made according to the surgeon’s experience and the type of injury [6,14].

Herbert screws, first used in the early 1990s to fix a scaphoid fracture, have become a successful tool to resolve the problems of irritation and motionlessness of K wires. We used headless screws in 4 cases, Herbert screws in 2 cases, and K wires in 5 cases for the fixation of scaphoid fractures. Though the application of headless screws is more difficult than K wires, if the fracture and surgical approach are suitable, headless screws are preferred for fixation [11]. We used different implants for fixation of the scaphoid, and no reduction loss or nonunion was observed. 

In all of our cases, we used a dorsal incision and Z capsulotomy, and then achieved reduction. In 1 case, a volar incision was also required for the reduction of the lunate. Accompanying triquetrum, radial styloid, and capitate fractures were fixed with K wires under fluoroscopy guidance. 

Various results have been reported in the surgical treatment of such injuries in the literature. Viegas et al. [15] used open reduction and osteosynthesis with Herbert screws in 5 cases of dorsal TSPFD, and reported 3 excellent, 1 good, and 1 moderate result in the clinical assessment. Ada et al. [4] applied open reduction, ligament repair, and K wires to fix fractures and joints in 6 cases and reported 1 excellent, 2 moderate, and 3 poor results according to the Green and O’Brien evaluation scale. In our study, 8 patients were treated in the acute phase and 3 patients were delayed, and we obtained 6 good, 3 moderate, and 2 poor results. 

In final postoperative controls, there was no correlation between the advanced degeneration stage and the range of motion, and there was a negative correlation between the VAS score and the wrist degeneration level. This suggests that the radiological view will not be effective alone in the assessment of a wrist complaint [1]. Komurcu et al. [3] reported that acute treatment had more successful results, although there was not always a correlation between the radiological and the clinical results. Ada et al. [4] evaluated their 8 perilunate fracture-dislocation cases and suggested that injury type, severity of the injury, type of perilunate dislocation, and time to diagnosis were important to the clinical and radiological results.

Postoperative cast immobilization might be needed, depending on additional pathologies. Although the duration of cast immobilization varies for each patient, it generally depends on the scaphoid fixation and intercarpal joint stabilization. In the literature, the length of time varies from 6 to 12 weeks. If the stabilization is good, it is recommended to limit the time in the cast to 6 weeks [11]. The cast was in place for 6 weeks for 7 of our patients, 8 weeks for 3, and 12 weeks for 1 patient. In the cases in which K wires were used to fix fractures, cast immobilization was applied for more than 6 weeks (Table 2).

Even though 3 of our cases were delayed cases, we found that after open reduction and stable fixation, the clinical and functional outcomes were similar to those of the acute phase cases.

The small number of cases and the absence of a comparison group are the weaknesses of our study.

In conclusion, this kind of injury involves complex biomechanical damage to the wrist anatomy. If TSPFD, which is especially observed in the young population, is diagnosed early and treated with open reduction and stable fixation, a functionally adequate and anatomically integrated wrist can be obtained.

## Acknowledgement

The authors declared no conflicts of interest with respect to the authorship and/or publication of this article. The authors received no financial support for the research and/or authorship of this article. Written informed consent was obtained from the patients for the publication of this article and for any accompanying images.

## References

[ref0] (2012). The functional results of treatment of perilunate dislocations with volar approach and K-wires fixation. Hand and Microsurgery.

[ref1] (2011). Perilunate injuries. Hand (NY).

[ref2] (2008). and delayed treatment of dorsal transscaphoid perilunate fracture-dislocations. Journal of Orthopaedic Trauma.

[ref3] (1995). Perilunate kırıklı-çıkıkların cerrahi tedavi sonuçları. Acta Orthopaedica et Traumatologica Turcica.

[ref4] (2007). Trans-triquetral dorsal perilunate fracture dislocation. Journal of Hand Surgery.

[ref5] (2002). Acute dorsal trans-scafoid perilunate fracture-dislocations: medium-term results. Journal of Hand Surgery (British and European.

[ref6] (1993). Perilunate dislocations and fracture-dislocations: a multicenter study. Journal of Hand Surgery (American.

[ref7] (1990). Treatment of trans-scafoid perilunate dislocations by internal fixation with the Herbert screw. Journal of Hand.

[ref8] (2012). Staged reduction of neglected transscafoid perilunate fracture dislocation: a report of 16 cases. Journal of Orthopaedic Surgery and Research.

[ref9] (1987). Difficult wrist fractures. Perilunate fracture-dislocations of the wrist. Clinical Orthopaedics and Related Research.

[ref10] (1988). Late treatment of a dorsal transscaphoid, transtriquetral perilunate wrist dislocation with avascular changes of the lunate. Clinical Orthopaedics and Related Research.

[ref11] (2010). Percutaneous fixation of selected scaphoid fractures. International Orthopaedics.

[ref12] (2008). Perilunate and axial carpal dislocations and fracture-dislocations. Journal of Hand Surgery (American.

[ref13] (1982). Treatment of acute lunate and perilunate dislocations. Clinical Orthopaedics and Related Research.

